# SQLE is a promising prognostic and immunological biomarker and correlated with immune Infiltration in Sarcoma

**DOI:** 10.1097/MD.0000000000037030

**Published:** 2024-02-09

**Authors:** Mengwei Shao, Mingbo Wang, Xiliang Wang, Xiaodong Feng, Lifeng Zhang, Huicheng Lv

**Affiliations:** a Department of Orthopedics, The Second Affiliated Hospital of Inner Mongolia Medical University, Hohhot, China

**Keywords:** immune infiltration, prognosis, sarcoma, Squalene epoxidase

## Abstract

Squalene epoxidase (SQLE) is an essential enzyme involved in cholesterol biosynthesis. However, its role in sarcoma and its correlation with immune infiltration remains unclear. All original data were downloaded from The Cancer Genome Atlas (TCGA). SQLE expression was explored using the TCGA database, and correlations between SQLE and cancer immune characteristics were analyzed via the TISIDB databases. Generally, SQLE is predominantly overexpressed and has diagnostic and prognostic value in sarcoma. Upregulated SQLE was associated with poorer overall survival, poorer disease-specific survival, and tumor multifocality in sarcoma. Mechanistically, we identified a hub gene that included a total of 82 SQLE-related genes, which were tightly associated with histone modification pathways in sarcoma patients. SQLE expression was negatively correlated with infiltrating levels of dendritic cells and plasmacytoid dendritic cells and positively correlated with Th2 cells. SQLE expression was negatively correlated with the expression of chemokines (CCL19 and CX3CL1) and chemokine receptors (CCR2 and CCR7) in sarcoma. In conclusion, SQLE may be used as a prognostic biomarker for determining prognosis and immune infiltration in sarcoma.

## 1. Introduction

Sarcoma is a rare and heterogeneous group of malignant tumors that arise from mesenchymal cells, including bone, cartilage, fat, and muscle. Sarcoma accounts for less than 1% of all adult cancers, and the overall incidence of sarcoma is about 5–6 cases per 100,000 people per year.^[1]^ Despite advances in diagnosis and treatment, the prognosis for patients with sarcoma remains poor, with a 5-year survival rate of less than 50%.^[2]^ The etiology of sarcomas is largely unknown, although certain environmental and genetic factors have been implicated in their development. At the molecular level, sarcomas are characterized by complex genetic and epigenetic alterations, which contribute to their pathogenesis and progression.^[3]^ The pathological and physiological mechanisms underlying sarcomagenesis are also complex and poorly understood. Currently, the diagnosis of sarcoma relies on a combination of imaging studies, histopathology, and molecular testing. Treatment options for sarcoma include surgery, radiation therapy, chemotherapy, and targeted therapy.^[4]^ However, the efficacy of these treatments is limited, and many patients experience relapse or disease progression. Therefore, there is a pressing need for a better understanding of the molecular and cellular mechanisms underlying sarcoma pathogenesis and for the development of novel therapeutic strategies.

Sarcomas are a group of rare malignancies that arise from mesenchymal tissues, and their prognosis remains poor despite advances in treatment modalities.^[4]^ Increasing evidence suggests that the tumor microenvironment (TME) plays a crucial role in tumor progression and response to therapy.^[5]^ In particular, immune cells infiltrating the TME have emerged as critical players in shaping tumor-immune interactions and can significantly impact patient outcomes. Among the immune cells, Th2 cells, dendritic cells (DCs), and plasmacytoid dendritic cells (pDCs) have been shown to have crucial functions in sarcoma pathogenesis. Th2 cells play a critical role in promoting tumor progression by modulating the immune response towards a pro-tumor state. DCs and pDCs have been implicated in both promoting and inhibiting tumor growth, depending on their activation state and the tumor context.^[6,7]^ Therefore, a better understanding of the interactions between immune cells and the TME may lead to the development of novel therapeutic strategies for sarcoma patients. In this study, we aimed to investigate the prognostic value of squalene epoxidase (SQLE) as a biomarker in sarcoma and explore its correlation with immune cell infiltration, focusing on the role of Th2 cells, DCs, and pDCs.

SQLE is a key enzyme involved in cholesterol biosynthesis, catalyzing the conversion of squalene to 2,3-oxidosqualene. The SQLE gene is located on chromosome 8q24.13 and encodes a protein with 593 amino acids. SQLE belongs to the family of flavin-containing monooxygenases and contains two domains: an *N*-terminal FAD-binding domain and a C-terminal oxygenase domain. SQLE is a conserved enzyme among eukaryotes and plays an essential role in maintaining cellular cholesterol homeostasis.^[8,9]^ In addition to its role in cholesterol biosynthesis, SQLE has been implicated in various cellular processes, including cell proliferation, migration, and apoptosis. The SQLE gene is also a member of the sterol regulatory element-binding protein (SREBP) pathway, which regulates cholesterol metabolism in response to cellular demand.^[10]^ Dysregulation of the SREBP pathway has been associated with various diseases, including metabolic disorders and cancer.^[11]^ SQLE has been implicated in the pathogenesis of several important diseases, including atherosclerosis and Alzheimer’s disease. Atherosclerosis is a chronic inflammatory disease of the arterial wall, characterized by the accumulation of lipids, inflammatory cells, and extracellular matrix components. Recent studies have shown that SQLE is upregulated in atherosclerotic lesions, and inhibition of SQLE activity can reduce the formation of atherosclerotic plaques in animal models.^[12,13]^ Alzheimer’s disease is a neurodegenerative disorder characterized by the accumulation of β-amyloid plaques and neurofibrillary tangles in the brain. SQLE has been shown to be involved in the regulation of β-amyloid production and neuroinflammation in Alzheimer’s disease.^[14,15]^ Recent studies have also demonstrated a significant correlation between SQLE expression and cancer progression. Overexpression of SQLE has been observed in several cancer types, including head and neck squamous cell carcinoma (HNSC), pancreatic adenocarcinoma, glioblastoma, colorectal cancer, and breast cancer (BRCA).^[16–20]^ In addition, SQLE has been shown to play a critical role in promoting cancer cell survival and proliferation by modulating cholesterol metabolism and lipid signaling pathways.^[10]^ In sarcoma, a rare type of cancer arising from connective tissues, the role of SQLE remains poorly understood.

In this study, we aimed to investigate the expression and clinical significance of SQLE in sarcoma using bioinformatics analysis of The Cancer Genome Atlas (TCGA) dataset. We also explored the potential biological functions of SQLE and its correlation with immune infiltrates in sarcoma. The results of this study may provide new insights into the molecular mechanisms underlying sarcoma and identify potential biomarkers and therapeutic targets for sarcoma treatment.

## 2. Material and methods

### 2.1. Patient datasets

The mRNA expression data, including 259 sarcoma samples and 2 adjacent nontumor samples (RNAseq and TPM), and clinical information were downloaded from the TCGA database (https://cancergenome.nih.gov). There was no requirement for ethics committee approval, and the data were extracted from the public database.

### 2.2. Survival analysis

Kaplan–Meier plots were generated, and the log-rank test was performed using sarcoma data from the TCGA database to estimate the correlation between SQLE expression and the survival rate of different clinical features in sarcoma patients. The correlation was estimated by the R survival package. The hazard ratio (HR) and log-rank *P* value of the 95% confidence interval were also calculated.^[21]^

### 2.3. Establishment and evaluation of the nomograms for sarcoma survival prediction

The pROC and time-receiver operating characteristic (ROC) R packages were used to create the ROC curve of diagnosis and the time-dependent curve of diagnosis. A nomogram was constructed by the R package to evaluate the 1-, 3-, and 5-year overall survival (OS) probability of sarcoma patients. Independent clinicopathological prognostic variables were chosen from Cox regression analysis.

### 2.4. Protein–Protein interaction network

The protein–protein interaction (PPI) data were extracted from the STRING database (https://string-db.org) based on protein interactions and signaling pathways to predict the PPI network of SQLE-coexpressed genes. An interaction with a combined score > .15 was considered statistically significant. The network was constructed using Cytoscape 3.9.1 applications.^[22]^

### 2.5. Functional enrichment analysis

The first 389 genes related to SQLE in sarcoma and the first 300 genes related to sarcoma survival were obtained from the TCGA. These genes were enriched by gene ontology (GO) (including biological processes [BP], cellular components [CC], and molecular function [MF]) and Kyoto Encyclopedia of Genes and Genomes (KEGG) pathway analyses using the Database for Annotation, Visualization and Integrated Discover (DAVID) and visualized by the R package ggplot2. Statistical significance was determined as a corrected *P* < .05.^[23]^

### 2.6. Immune infiltration analysis by single-sample GSEA

The GSVA package in R (http://www.biocondutor.org/package/release/bioc/html/GSVA.html) was used to perform immune infiltration analysis of sarcoma samples by the single-sample gene set enrichment analysis (ssGSEA) method for 24 types of immune cells. Spearman’s correlation coefficient analysis was performed to identify relationships between sarcoma and each immune cell subset.

### 2.7. LinkedOmics database analysis

The LinkedOmics database (http://www.linkedomics.org) was used to explore the expression profile of SQLE. The GSEA in the Link Interpreter module was used to explore the GO and KEGG pathways of SQLE and its coexpressed genes.^[24]^

### 2.8. TISIDB database analysis

The TISIDB database (http://cis.hku.hk/TISIDB/) was used to analyze and evaluate the correlation between SQLE mRNA expression levels and the levels of immune checkpoint genes by using the “Immunomodulator” module. The “chemokine” module was used to study the association between SQLE mRNA expression levels and chemokine/chemokine receptor expression.^[25]^

### 2.9. Statistical analysis

For statistical analysis, box plots were used to assess the expression level of the SQLE gene in sarcoma patients, with the median method of gene expression being used as the cutoff value of SQLE expression. The Wilcoxon signed-rank test was utilized to investigate the relationship between clinical characteristics and SQLE expression in sarcoma. Univariate Cox analysis was conducted to test for possible prognostic factors, while multivariate Cox analysis was used to confirm the influence of SQLE expression on survival in conjunction with other clinical variables. All statistical analyses were performed using R statistical software (version 3.5.3) or SPSS software (version 24.0), and a *P* value of less than .05 was considered statistically significant.

## 3. Results

### 3.1. Baseline characteristics of patients

Data from a total of 259 sarcoma patients with the required clinical features were acquired from the TCGA data portal. The detailed clinical features are listed in Table 1. Among the 259 participants, 118 were male (45.5%) and 141 were female (54.5%). Among them, 128 patients (49.4%) were younger than or equal to 60 years, and 131 patients (50.6%) were older than 60 years. In terms of histological type, 9 patients had malignant peripheral nerve sheath tumors (3.5%), 58 patients had dedifferentiated liposarcoma (22.4%), 114 patients had leiomyosarcoma (40.1%), 51 patients had pleomorphic sarcoma (19.7%), 25 patients had myxofibrosarcoma (9.6%), and 12 patients had desmoid tumor and synovial sarcoma (4.7%). In terms of residual tumor, 154 patients had R0 (66.4%), 69 patients had R1(29.8%), and 9 patients had R2 (3.9%). Meanwhile, 197 patients had nonmultifocal lesion (83.1%) and 40 had multifocal lesion (16.9%). In terms of tumor necrosis, 70 patients had no necrosis (38.6%), 38 patients had focal necrosis (20.9%), 61 patients had moderate necrosis (33.7%), 12 patients had no necrosis (6.7%). As to tumor depth, 21 patients were superficial (10.2%) and 185 patients were deep (89.8%). Furthermore, 120 patients had no metastases (68.2%) and 26 had metastases (31.8%); only 76 received radiation therapy (30.0%) and the other 177 patients did not (70.0%). In overall survival, 161 patients (62.2%) were still alive and 98 (37.8%) had died; during disease-specific survival, 172 patients (68.0%) were still alive, but 81 died (32.0%) from the disease; in the disease-free interval progression, 121 patients (46.7%) had no disease progression and 138 patients (53.3%) had disease progression (Table 1).

**Table 1:** Clinical characteristics of the sarcoma cell carcinoma patients.

Characteristics	Low expression of SQLE	High expression of SQLE	*P* value
n	129	130	
Gender, n (%)			.421
Female	67 (25.9%)	74 (28.6%)	
Male	62 (23.9%)	56 (21.6%)	
Race, n (%)			.698
Asian & Black or African American	13 (5.2%)	11 (4.4%)	
White	113 (45.2%)	113 (45.2%)	
Age, n (%)			.757
≤60	65 (25.1%)	63 (24.3%)	
>60	64 (24.7%)	67 (25.9%)	
Histological type, n (%)			.031
Malignant peripheral nerve sheath tumors	3 (1.2%)	6 (2.3%)	
Dedifferentiated liposarcoma	23 (8.9%)	35 (13.5%)	
Leiomyosarcoma	57 (22%)	47 (18.1%)	
Pleomorphic sarcoma	25 (9.7%)	26 (10%)	
Myxofibrosarcoma	18 (6.9%)	7 (2.7%)	
Desmoid tumor & synovial sarcoma	3 (1.2%)	9 (3.5%)	
Residual tumor, n (%)			.093
R0	84 (36.2%)	70 (30.2%)	
R1	31 (13.4%)	38 (16.4%)	
R2	2 (0.9%)	7 (3%)	
Tumor multifocal, n (%)			.012
No	107 (45.1%)	90 (38%)	
Yes	13 (5.5%)	27 (11.4%)	
Tumor necrosis, n (%)			.901
No necrosis	35 (19.3%)	35 (19.3%)	
Focal necrosis	18 (9.9%)	20 (11%)	
Moderate necrosis	32 (17.7%)	29 (16%)	
Extensive necrosis	5 (2.8%)	7 (3.9%)	
Tumor depth, n (%)			.213
Superficial	13 (6.3%)	8 (3.9%)	
Deep	88 (42.7%)	97 (47.1%)	
Metastasis, n (%)			.082
No	64 (36.4%)	56 (31.8%)	
Yes	22 (12.5%)	34 (19.3%)	
Margin status, n (%)			.243
Negative	73 (34.9%)	63 (30.1%)	
Positive	33 (15.8%)	40 (19.1%)	
Radiation therapy, n (%)			.880
No	88 (34.8%)	89 (35.2%)	
Yes	37 (14.6%)	39 (15.4%)	
OS event, n (%)			<.001
Alive	96 (37.1%)	65 (25.1%)	
Dead	33 (12.7%)	65 (25.1%)	
DSS event, n (%)			<.001
No	99 (39.1%)	73 (28.9%)	
Yes	28 (11.1%)	53 (20.9%)	
PFI event, n (%)			.093
No	67 (25.9%)s	54 (20.8%)	
Yes	62 (23.9%)	76 (29.3%)	

OS = overall survival, SQLE = squalene epoxidase.

### 3.2. SQLE is highly expressed in sarcoma

**Figure 1.**
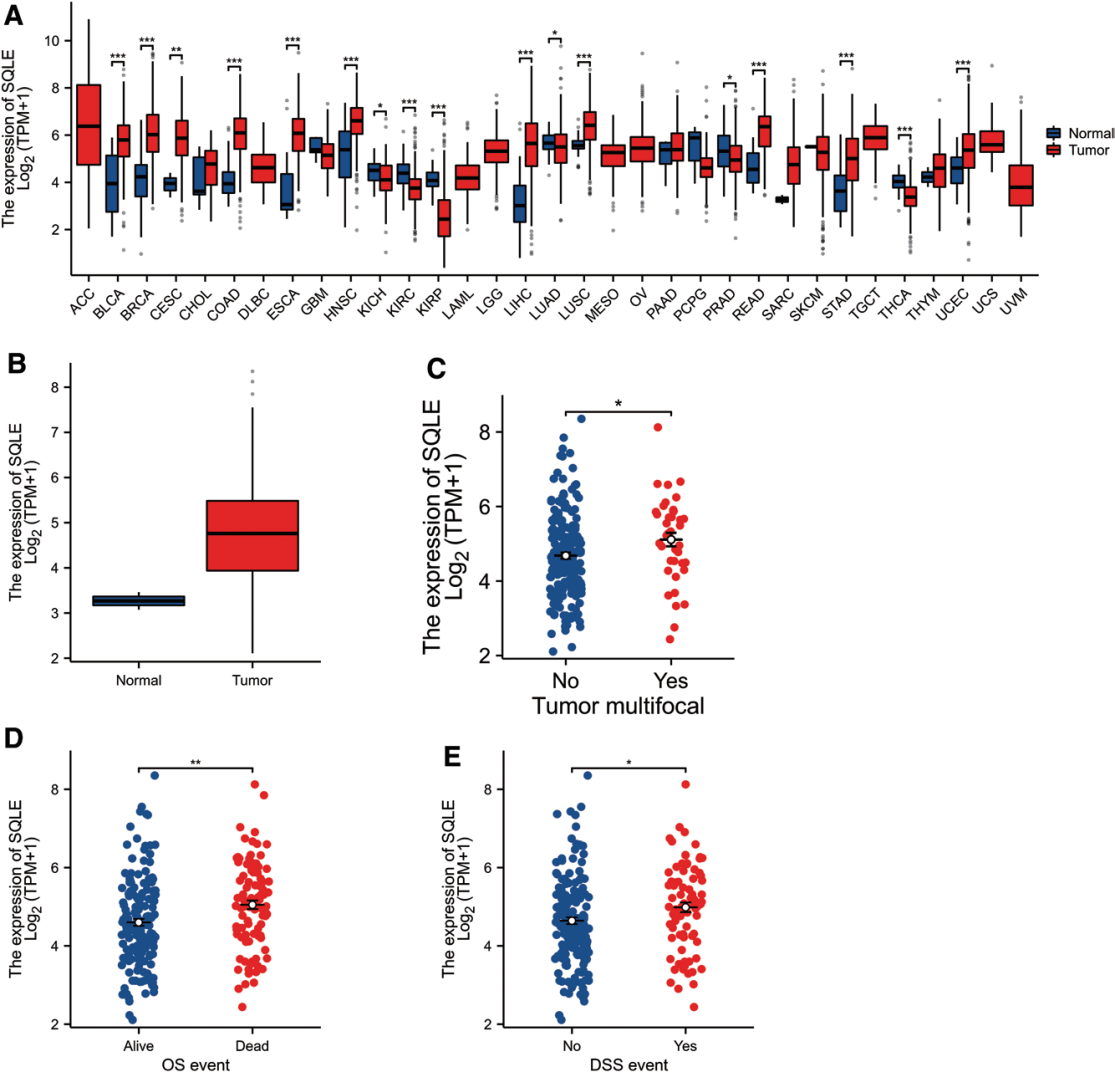
The expression level of SQLE in different human cancers. (A) Increased or decreased SQLE expression in datasets of different cancers compared with normal tissues in the TCGA database. (B) Expression level of SQLE in normal tissues and unmatched tumor tissues. (C–E) Expression level of SQLE in normal tissues and unmatched tumor tissues from patients with different clinical characteristics in TCGA [Tumor multifocal (C), OS event (D), and DSS event (E)]. *P < .05, **P < .01, ***P < .001. SQLE = squalene epoxidase, TCGA = The Cancer Genome Atlas.

To explore the expression level of SQLE in normal and tumor tissues, we downloaded and analyzed the expression levels of SQLE mRNA in different tumors and normal tissues from the TCGA using the R package. The results showed that SQLE expression was significantly higher in tumor tissues such as bladder urothelial carcinoma, BRCA, cervical squamous cell carcinoma, colon adenocarcinoma, esophageal carcinoma, HNSC, kidney chromophobe, kidney renal clear cell carcinoma, kidney renal papillary cell carcinoma, liver hepatocellular carcinoma, lung adenocarcinoma, lung squamous cell carcinoma, prostate adenocarcinoma, rectum adenocarcinoma, stomach adenocarcinoma, thyroid carcinoma and uterine corpus endometrial carcinoma (Fig. 1A). The expression level of SQLE in sarcoma tended to be higher than that in normal stroma, but because the number of samples in normal stroma was too small, the difference between the 2 groups could not be analyzed by statistical methods (Fig. 1A,B). To analyze the correlation between SQLE expression and clinical characteristics in sarcoma patients, we analyzed the mRNA expression levels of SQLE in different clinical categories in the TCGA database. The results showed that high expression of SQLE was significantly related to tumor multifocal (*P* < .05), OS event (*P* < .05), and DSS event (*P* < .05) (Fig. 1C–E). These data suggest that SQLE is upregulated in sarcoma.

### 3.3. High expression of SQLE is an independent prognostic factor for the overall survival of sarcoma

**Figure 2.**
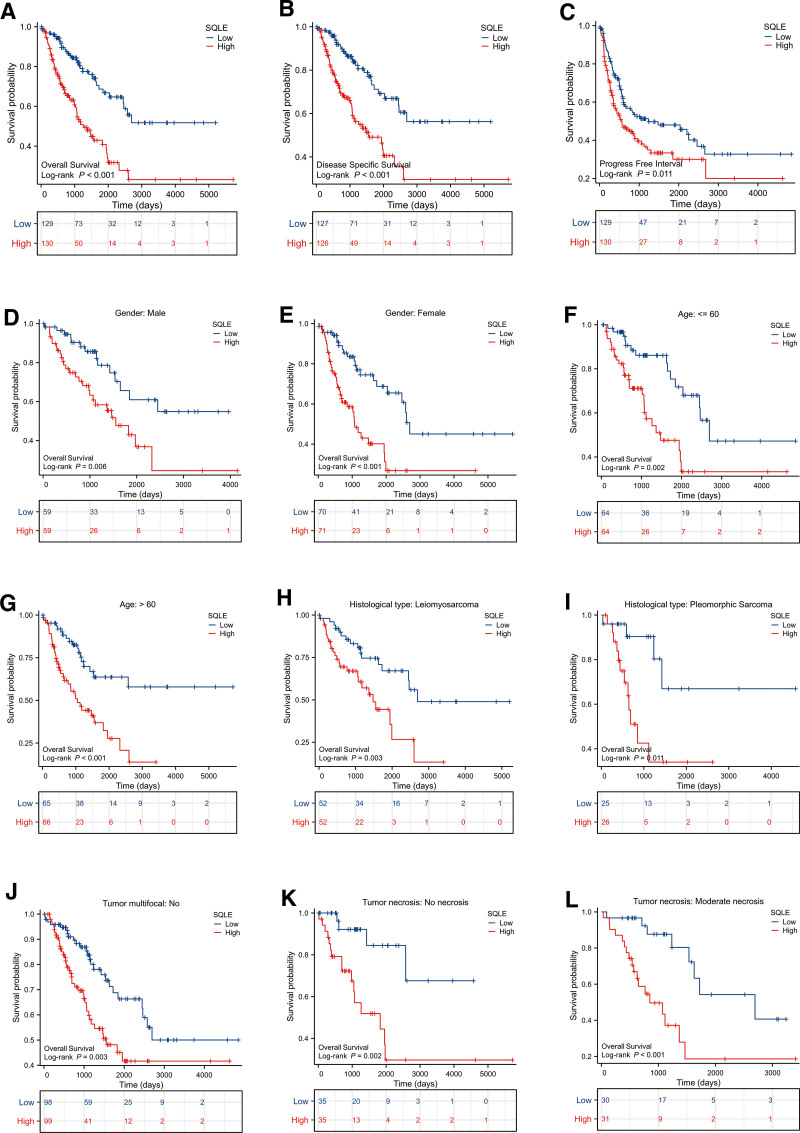
Kaplan–Meier survival curve analysis of the prognostic significance of a high and a low expression of SQLE in sarcoma using The Cancer Genome Atlas (TCGA). (A) Kaplan–Meier estimates of the overall survival probability of TCGA patients in all sarcoma patients. (B–C) Kaplan–Meier estimates of the disease-specific survival (B) and progression-free interval (C) probability of TCGA patients in all sarcoma patients. (D–L) Subgroup analysis for male (D), female (E), ≤60 years old (F), >60 years old (G), leiomyosarcoma (H), pleomorphic sarcoma (I), no tumor multifocal (J), no necrosis (K), moderate necrosis (L). SQLE = squalene epoxidase.

**Figure 3.**
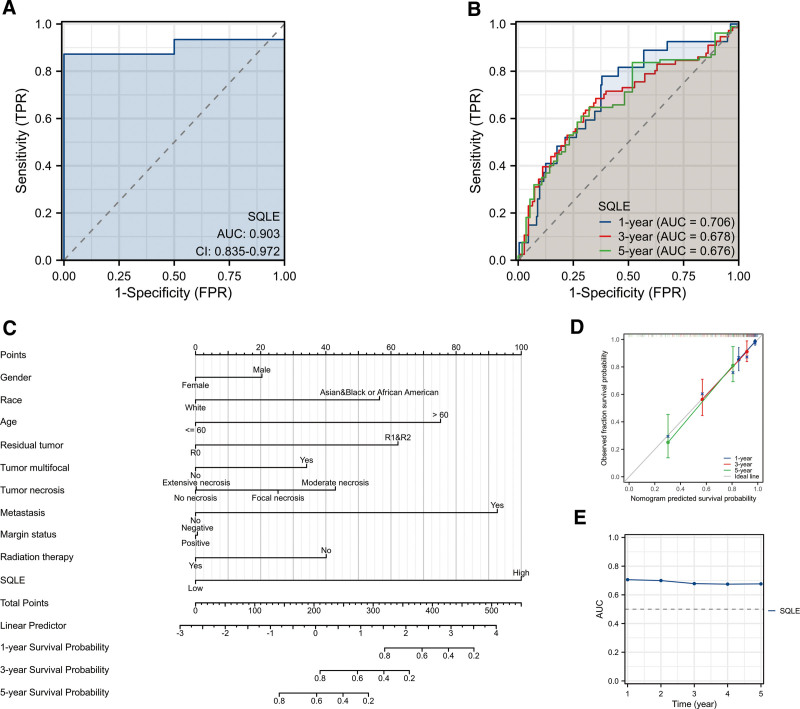
Diagnostic value of SQLE expression in sarcoma. (A) Receiver operating characteristic (ROC) curve analysis for SQLE expression in sarcoma and adjacent tissue. (B) Time-dependent survival ROC analysis for SQLE expression in sarcoma and adjacent tissue to predict 1-, 3- and 5-year survival rates. (C) Nomogram survival prediction chart for predicting the 1-, 3-, and 5-year overall survival (OS) rates. (D) Calibration plots of the nomogram for predicting the probability of OS at 1, 3, and 5 years. (E)Time-dependent AUC curve analysis for SQLE expression in sarcoma and adjacent tissue. AUC = area under the receiver operating characteristic curve, SQLE = squalene epoxidase.

To identify whether SQLE expression affects patient survival, we classified sarcoma patients in the TCGA database into a high SQLE expression group (the top 50% of samples with the highest expression) and a low SQLE expression group (the remaining 50% of the samples) to perform survival analysis according to the mean expression value of SQLE. The Kaplan–Meier survival analysis showed that the high expression of SQLE was related to the poor prognosis in overall survival (*P* < .001), disease-specific survival (*P* < .001), and progression-free interval (*P* = .011) of sarcoma patients (Fig. 3A–C). Subgroup analysis showed that high SQLE expression was significantly correlated with poor prognosis in sarcoma in the following cases: male (*P* = .006), female (*P* < .001), age ≤ 60 years old (*P* = .002), age >60 years old (*P* < .001), histological type: leiomyosarcoma (*P* = .003), histological type: pleomorphic sarcoma (*P* = .011), no tumor multifocal (*P* = .003), no tumor necrosis (*P* = .002) and tumor moderate necrosis (*P* < .001) (Fig. 2, Table 2). These data suggest that high expression of SQLE is an independent prognostic factor for the OS of sarcoma patients.

**Table 2:** Univariate and multivariate Cox regression analyses of clinical characteristics associated with overall survival.

Characteristics	Total (N)	Univariate analysis		Multivariate analysis	
		Hazard ratio (95% CI)	*P* value	Hazard ratio (95% CI)	*P* value
Gender	259		.442		
Female	141	Reference			
Male	118	0.855 (0.572–1.277)	.443		
Race	250		.429		
Asian & Black or African American	24	Reference			
White	226	0.736 (0.356–1.525)	.410		
Age	259		.135		
≤60	128	Reference			
>60	131	1.354 (0.908–2.019)	.137		
Histological type	259		.767		
Leiomyosarcoma	104	Reference			
Dedifferentiated liposarcoma	58	1.187 (0.721–1.953)	.500		
Pleomorphic sarcoma	51	1.070 (0.599–1.911)	.820		
Desmoid tumor & myxofibrosarcoma & malignant peripheral nerve sheath tumors & synovial sarcoma	46	0.852 (0.478–1.519)	.588		
Residual tumor	232		**<.001**		
R0	154	Reference		Reference	
R1 & R2	78	2.554 (1.668–3.910)	**<.001**	1.843 (0.719–4.726)	.203
Tumor multifocal	237		**<.001**		
No	197	Reference		Reference	
Yes	40	2.404 (1.502–3.847)	**<.001**	1.125 (0.448–2.825)	.802
Tumor necrosis	181		.358		
No necrosis	70	Reference			
Focal necrosis	38	1.481 (0.747–2.938)	.261		
Moderate necrosis	61	1.672 (0.931–3.001)	.085		
Extensive necrosis	12	1.461 (0.546–3.911)	.450		
Tumor depth	206		**.031**		
Superficial	21	Reference		Reference	
Deep	185	2.920 (0.920–9.274)	.069	2.963 (0.391–22.436)	.293
Metastasis	176		**<.001**		
No	120	Reference		Reference	
Yes	56	3.014 (1.834–4.954)	**<.001**	2.514 (1.395–4.531)	**.002**
Margin status	209		**.015**		
Negative	136	Reference		Reference	
Positive	73	1.840 (1.138–2.974)	**.013**	1.047 (0.427–2.567)	.919
Radiation therapy	253		.468		
No	177	Reference			
Yes	76	0.850 (0.545–1.325)	.473		
SQLE	259		**<.001**		
Low	129	Reference		Reference	
High	130	2.725 (1.785–4.161)	**<.001**	3.146 (1.567–6.318)	**.001**

CI = confidence interval, SQLE = squalene epoxidase.

### 3.4. Diagnostic value of SQLE expression in sarcoma

To analyze the diagnostic value of SQLE expression in sarcoma, we performed ROC curve and nomogram analyses on the SQLE gene expression data from the TCGA database to evaluate the diagnostic value of the gene. The area under the ROC curve (AUC) was 0.901, suggesting a higher diagnostic value, as shown in Figure 3A. A time-dependent survival ROC curve of SQLE was created to predict the 1-, 3-, and 5-year survival rates. All these AUC values were above 0.60, indicating that the model had moderate predictive value in predicting 1, 3, and 5-year survival rates^[26]^ (Figure 3B). Then, we combined the expression level of SQLE with the clinical variables to construct a nomogram to predict the survival probability of patients at 1, 3, and 5 years. The nomogram indicated that the prognostic prediction of the expression level of SQLE was better than that of the traditional clinical features of age, gender, residual tumor, tumor multifocal, radiation therapy, and margin status (Fig. 3C). The result of the calibration curve demonstrated that the predictive probability of 1-, 3-, and 5-year survival was close to the actual 1-year survival in the clinic (Fig. 3D,E).

**Table 3:** Gene sets enriched in the SQLE-related genes (top 389).

Ontology	ID	Description	GeneRatio	BgRatio	*P* value	*P* adjust
BP	GO:0022613	Ribonucleoprotein complex biogenesis	35/366	448/18800	2.97e-12	9.56e-09
BP	GO:0042254	Ribosome biogenesis	27/366	304/18800	5.7e-11	8.18e-08
BP	GO:0016072	rRNA metabolic process	25/366	264/18800	7.63e-11	8.18e-08
BP	GO:0006364	rRNA processing	22/366	226/18800	6.36e-10	5.12e-07
BP	GO:0006888	Endoplasmic reticulum to Golgi vesicle-mediated transport	15/366	129/18800	3.29e-08	2.12e-05
CC	GO:0030684	Preribosome	13/381	78/19594	3.26e-09	1.42e-06
CC	GO:0140534	Endoplasmic reticulum protein-containing complex	14/381	125/19594	1.51e-07	3.29e-05
CC	GO:0032040	Small-subunit processome	8/381	39/19594	6.9e-07	8.46e-05
CC	GO:0030176	Integral component of endoplasmic reticulum membrane	15/381	164/19594	7.78e-07	8.46e-05
CC	GO:0042470	Melanosome	12/381	109/19594	1.41e-06	8.86e-05
MF	GO:0030515	snoRNA binding	9/376	32/18410	1.04e-08	6.02e-06
MF	GO:0016887	ATP hydrolysis activity	22/376	325/18410	1e-06	.0003
MF	GO:0043021	Ribonucleoprotein complex binding	14/376	150/18410	2.51e-06	.0005
MF	GO:0004386	Helicase activity	14/376	155/18410	3.69e-06	.0005
MF	GO:0140098	Catalytic activity, acting on RNA	22/376	388/18410	1.73e-05	.0018
KEGG	hsa04141	Protein processing in endoplasmic reticulum	17/174	171/8164	1.16e-07	2.69e-05
KEGG	hsa03008	Ribosome biogenesis in eukaryotes	10/174	109/8164	0.0001	0.0120
KEGG	hsa00920	Sulfur metabolism	3/174	10/8164	.0010	.0534
KEGG	hsa01250	Biosynthesis of nucleotide sugars	5/174	37/8164	.0010	.0534
KEGG	hsa03040	Spliceosome	10/174	147/8164	.0011	.0534

KEGG = Kyoto Encyclopedia of Genes and Genomes.

### 3.5. Functional enrichment and analyses of SQLE-related genes in sarcoma

**Figure 4.**
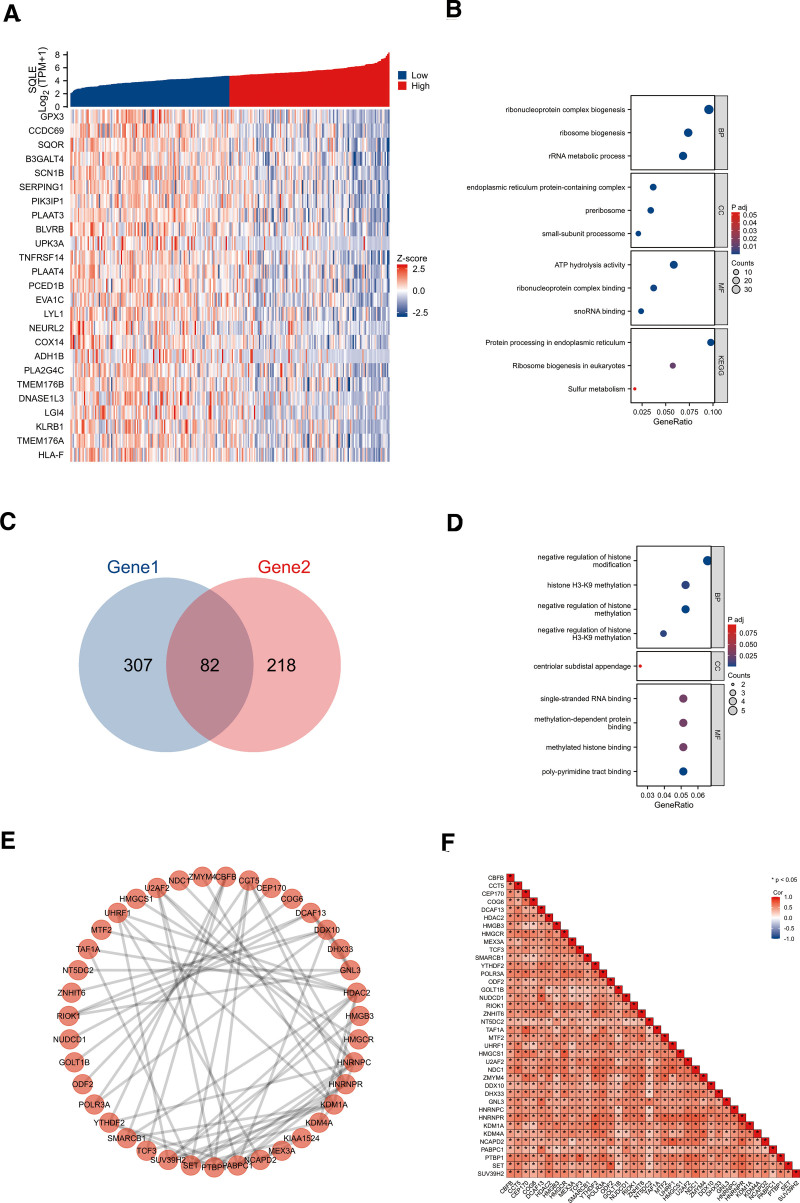
SQLE functional clustering and interaction network analysis of sarcoma-related genes. (A) Heatmap showing the top 50 genes in sarcoma that were positively and negatively related to SQLE. Red represents positively related genes and blue represents negatively related genes. (B) Gene Ontology (GO) term and Kyoto Encyclopedia of Genes and Genomes (KEGG) pathway analyses of SQLE-related genes in sarcoma. (C) Venn diagram of the SQLE-related genes and the survival-related genes in sarcoma. (D) GO term and KEGG pathway analyses of the SQLE-related genes and survival-related genes in sarcoma. (E) Both SQLE-related genes and the survival-related genes interaction network. (F) Gene co-expression matrix. SQLE = squalene epoxidase.

To understand the biological function of SQLE in sarcoma, we used the LinkFinder module of the LinkedOmics website to detect the co-expression pattern of SQLE in sarcoma in the TCGA database. The red dot indicates the top 25 genes that were positively correlated with SQLE, and the green dot represents the bottom 25 genes that were negatively correlated with SQLE (Fig. 4A). We used DAVID Functional Annotation Bioinformatics Microarray Analysis to identify the enriched GO functional enrichment and KEGG pathways among the SQLE-related 389 genes (the absolute value of correlation coefficient was >0.45) and found that ribonucleoprotein complex biogenesis, ribosome biogenesis and rRNA metabolic process were enriched among these genes (Fig. 4B and Table 3). To identify genes with the same regulatory direction in high SQLE and nonsurvival patients, we crossed the 389 genes with the highest SQLE correlation with the 300 survival-related upregulated genes (p. cox < 0.05) in sarcoma and detected 82 genes at the intersection that were related to SQLE and sarcoma survival (Fig. 4C). These 82 protein-coding genes may be potential genetic biomarkers for sarcoma patients. GO functional enrichment and KEGG pathway analysis were performed for these 82 genes, and the results showed that differentially expressed genes (DEGs) were significantly enriched in negative regulation of histone modification, histone H3-K9 mRNA expression, and negative regulation of histone methylation (Fig. 4D and Table 4). After discovering significantly different pathways, PPIs and correlated analysis were used to identify the interactions between these 82 proteins. We found that there was a stronger enrichment network among these proteins than in random proteins (Fig. 4E). Gene co-expression correlation analysis showed that most of the proteins in the network had a strong positive correlation with each other (Fig. 4F). Therefore, these SQLE-associated established genes have a strong intertwined interaction and can be used as multigene biomarkers to predict the survival of sarcoma patients.

**Table 4:** Gene sets enriched in the 82 genes at the intersection that were related to SQLE and sarcoma survival.

Ontology	ID	Description	GeneRatio	BgRatio	*P* value	*P* .adjust
BP	GO:0031057	Negative regulation of histone modification	5/76	50/18800	1.74e-06	.0013
BP	GO:0031061	Negative regulation of histone methylation	4/76	23/18800	2.06e-06	.0013
BP	GO:0051573	negative regulation of Histone H3-K9 methylation	3/76	12/18800	1.36e-05	.0057
BP	GO:0051567	Histone H3-K9 methylation	4/76	39/18800	1.82e-05	.0058
BP	GO:0018205	Peptidyl-lysine modification	9/76	392/18800	2.82e-05	.0071
CC	GO:0120103	Centriolar subdistal appendage	2/78	10/19594	.0007	.0938
MF	GO:0008187	Poly-pyrimidine tract binding	4/78	28/18410	5.65e-06	.0014
MF	GO:0035064	Methylated histone binding	4/78	78/18410	.0003	.0274
MF	GO:0140034	Methylation-dependent protein binding	4/78	78/18410	.0003	.0274
MF	GO:0003727	Single-stranded RNA binding	4/78	87/18410	.0005	.0274
MF	GO:0031492	Nucleosomal DNA binding	3/78	40/18410	.0006	.0274

### 3.6. Correlation of SQLE expression with immune characteristics

**Figure 5.**
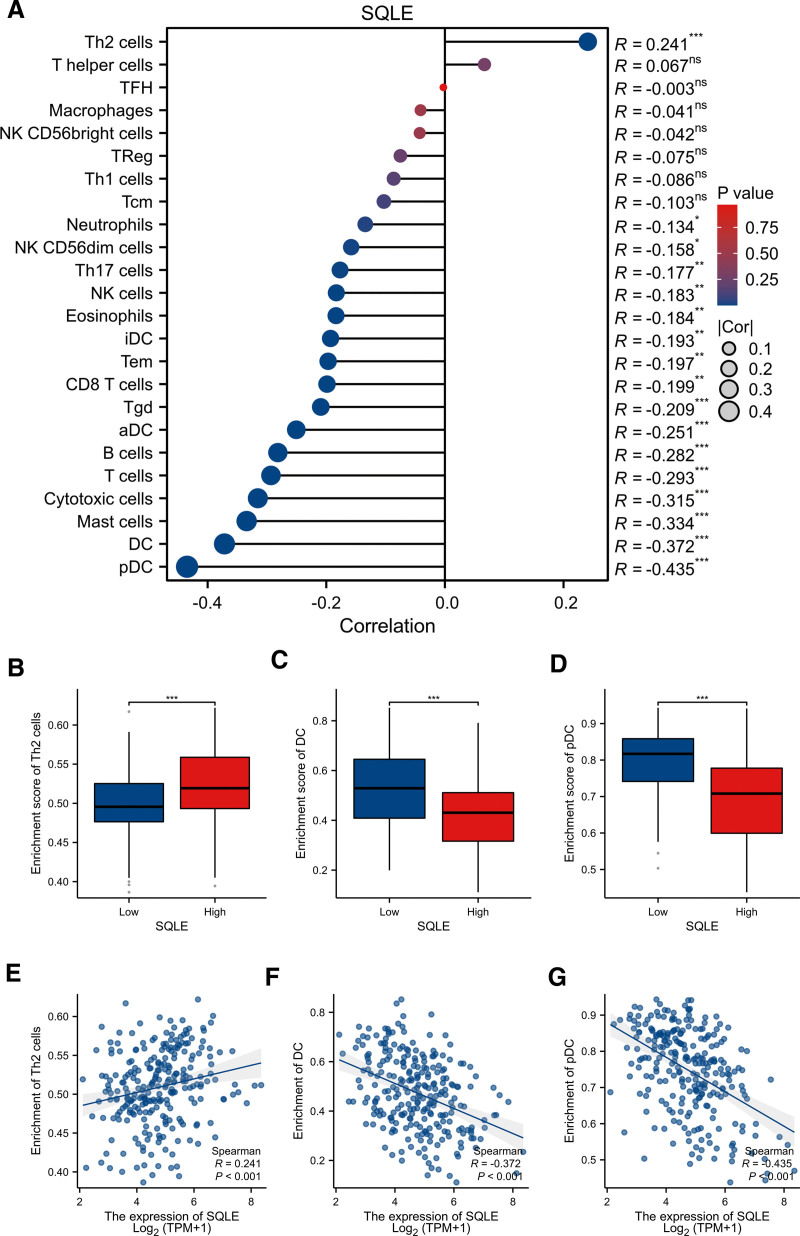
The expression level of SQLE was associated with immune infiltration in the tumor microenvironment. (A) Correlation between the relative abundances of 24 immune cells and SQLE expression level. The size of dots shows the absolute value of Spearman R. (B–G) Scatter plots and correlation diagrams showing the difference of Th2 cells, dendritic cells (DC), and plasmacytoid dendritic cells (pDC) infiltration level between SQLE-high and -low groups. SQLE = squalene epoxidase.

**Figure 6.**
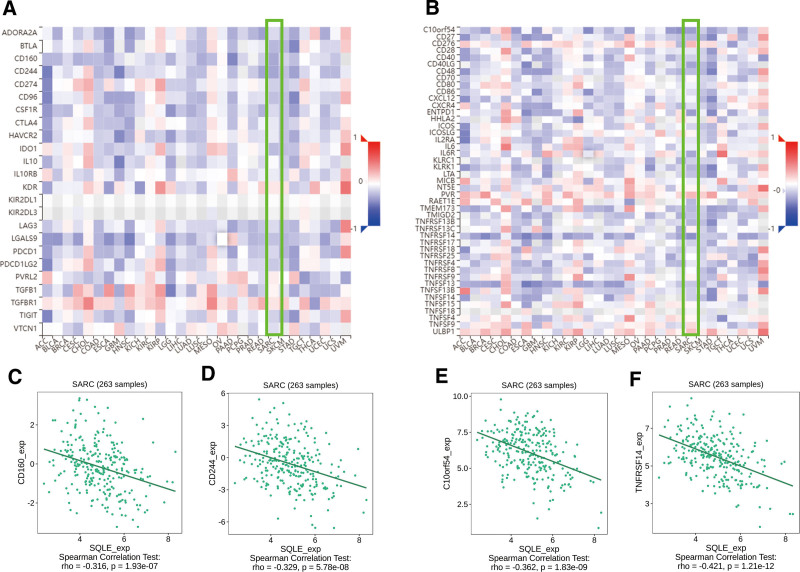
Correlation analysis between SQLE mRNA expression and immunoinhibitors and immunostimulators. (A) Correlation between SQLE and immunoinhibitors in tumors by heatmap analysis. (B) Correlation between SQLE and immunostimulators in tumors by heatmap analysis. (C–F) SQLE mRNA expression in sarcoma is negatively correlated with CD160, CD244, C10orf54, and TNFRSF14. SQLE = squalene epoxidase.

**Figure 7.**
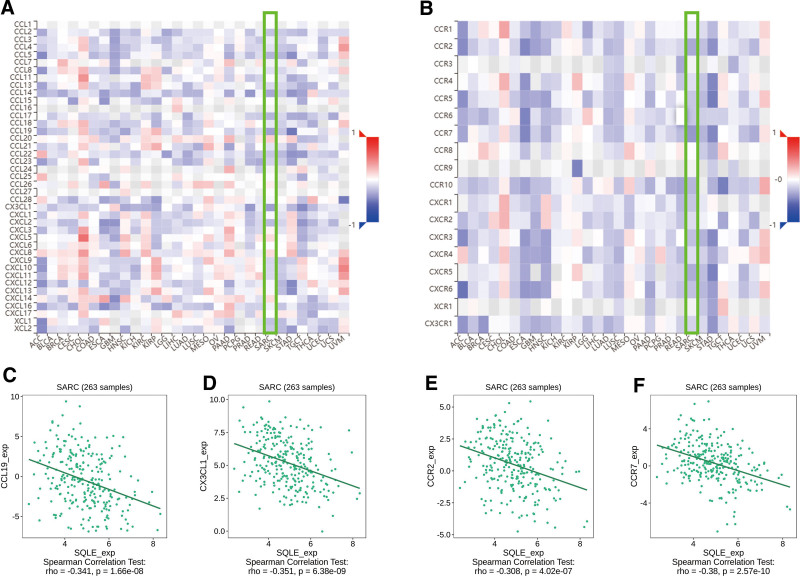
Correlation analysis between SQLE mRNA expression and chemokines and/or chemokine receptors. (A) Correlation between SQLE and chemokines in tumors by heatmap analysis. (B) Correlation between SQLE and chemokine receptors in tumors by heatmap analysis. (C–F) SQLE mRNA expression in sarcoma is negatively correlated with CCL19, CX3CL1, CCR2, and CCR7. SQLE = squalene epoxidase.

The correlation between the expression level (TPM) of SQLE and immune cell infiltration level quantified by ssGSEA was analyzed by Spearman correlation. The expression of SQLE was negatively correlated with dendritic cells (*R* = −0.372; *P <* .001), plasmacytoid dendritic cells (*R* = −0.435; *P <* .001) and positively correlated with the Th2 cells (*R* = 0.241; *P* < .001) (Fig. 5A–G). Therefore, we used TISIDB to analyze the correlation between the mRNA expression level of SQLE and immune cell chemokines and chemokine receptors in sarcoma. The heatmap results showed that several chemokines and chemokine receptors were significantly correlated with the mRNA expression level of SQLE in sarcoma (Fig. 6A,B). These results demonstrate that SQLE mRNA expression may play an important role in tumor immunity. To further clarify the relationship between SQLE mRNA expression and immune cell migration, we comprehensively analyzed the correlation between SQLE mRNA expression and chemokine/chemokine receptors. The results showed that SQLE mRNA expression was negatively correlated with CD160 (*R* = −0.316; *P <* .001), CD244 (*R* = −0.329; *P <* .001), C10orf54 (*R* = −0.362; *P <* .001), and TNFRSF14 (*R* = −0.421; *P <* .001) (Fig. 6C–F). These data indicated that SQLE mRNA expression is positively correlated with the expression of chemokines/chemokine receptors in sarcoma. Immune checkpoint inhibitors are a significantly novel strategy for tumor immunotherapy that has gradually improved the prognosis of patients with many types of cancers. Subsequently, we analyzed the correlation between SQLE mRNA expression and the expression of immunoinhibitors and immunostimulators in different types of human cancers using the TISIDB database (Fig. 7A,B). Interestingly, SQLE mRNA expression was negatively correlated with CCL19 (*R* = −0.341; *P <* .001), CX3CL1 (*R* = −0.351; *P <* .001), CCR2 (*R* = −0.308; *P <* .001), and CCR7 (*R* = −0.38; *P <* .001) (Fig. 7C–F). Therefore, these results suggest that SQLE may play a role in regulating tumor immunity.

## 4. Discussion

The SQLE gene encodes an enzyme called squalene monooxygenase, which is involved in the biosynthesis of cholesterol. Squalene monooxygenase catalyzes the conversion of squalene to 2,3-oxidosqualene, which is a key step in the cholesterol synthesis pathway. This pathway is crucial for many biological processes in the body, including the production of hormones and cell membrane structure.^[9,27]^ Mutations in the SQLE gene have been linked to a rare genetic disorder called lathosterolosis, which is characterized by high levels of the intermediate compound lathosterol and low levels of cholesterol. This disorder affects the normal development and function of many organs, including the brain, liver, and kidneys.^[28]^ Research suggests that SQLE may play a role in cancer development and progression. For example, studies have shown that SQLE is overexpressed in certain types of cancer, including HNSC, pancreatic adenocarcinoma, glioblastoma, colorectal cancer, and BRCA.^[16–20]^ In these cancers, SQLE may contribute to the growth and survival of cancer cells by promoting cholesterol biosynthesis and altering signaling pathways.^[29]^ However, there are no reports of SQLE in sarcoma. We integrated multiple bioinformatic analysis methods to determine its biological functions and potential regulatory pathways in sarcoma. In this study, we first determined the expression and prognostic value of the SQLE gene in cancer and found that the expression level of SQLE was upregulated in sarcoma. High SQLE expression was associated with poorer OS in sarcoma. Meanwhile, the high SQLE expression level was associated with tumor multifocal, poorer OS, and disease-specific survival. In addition, we also found that SQLE has an important reference value for the diagnosis of sarcoma. These findings strongly suggest that SQLE can be used as a biomarker for sarcoma diagnosis and prognosis.

To explain the underlying molecular mechanism by which SQLE affects sarcoma prognosis, we defined 82 genes in the SQLE gene network as our hub genes, which were 389 genes closely associated with sarcoma prognosis and 300 genes with the highest correlation with SQLE intersection. Then we further analyzed the biological process and signaling pathway of hub genes using GO functional enrichment and the KEGG pathway. The enrichment results suggested that most of the hub genes were related to biological processes such as histone H3-K9 methylation. H3-K9 methylation is a type of epigenetic modification that occurs on a specific amino acid residue (lysine 9) of the histone H3 protein.^[30]^ Histones are proteins that help package DNA into a compact structure called chromatin, which plays a critical role in regulating gene expression.^[31]^ Methylation of H3-K9 is catalyzed by a group of enzymes called histone methyltransferases. This modification typically results in the compaction of chromatin and the silencing of nearby genes. Dysregulation of H3-K9 methylation has been implicated in various diseases, including cancer and neurodevelopmental disorders, such as synovial sarcoma and malignant peripheral nerve sheath tumors.^[32]^ Therefore, SQLE may be involved in regulating sarcoma progression by regulating H3-K9 methylation.

A large number of studies have pointed out that the development of cancer is closely related to TME. The TME is composed of immune cells, extracellular matrix, and inflammatory mediators, which interact with each other to promote the growth, survival, and metastasis of tumor cells. Although an effective immune response can produce antitumor effects, cancer cells can evade attack by immune cells through antigen-presentation dysfunction and recruitment of immunosuppressive cells. Previous studies have reported that the degree of immune cell infiltration in tumors can affect the prognosis of patients, and the grade of tumor-infiltrating lymphocytes is an independent predictor of the prognosis of tumor patients.^[33,34]^ Our results showed that the expression of SQLE was negatively correlated with the infiltration level of dendritic cells and plasmacytoid dendritic cells and positively correlated with Th2 cells. Th2 cells are a type of T helper cell that promotes humoral immunity and is associated with allergy and asthma. In the context of cancer, Th2 cells have been shown to promote tumor growth and metastasis by suppressing the anti-` response.^[6]^ Therefore, the increase in Th2 cells in the TME with the increase of SQLE expression may facilitate tumor progression. DCs are antigen-presenting cells that play a critical role in initiating and regulating the adaptive immune response. pDCs are a specialized type of DCs that produce type I interferon in response to viral infection. Both DCs and pDCs are involved in the recognition and elimination of tumor cells.^[35]^ The decrease in the number of DCs and pDCs in the TME with the increase of SQLE expression may contribute to the immunosuppressive environment and promote tumor growth and metastasis.^[36]^ These findings suggest that SQLE may play an important regulatory role in the tumor immune microenvironment and sarcoma development.

Immunostimulators are substances that boost the immune system’s response to cancer cells. These substances can include cytokines, which are signaling molecules that stimulate the immune system, and monoclonal antibodies, which are antibodies designed to target specific proteins in cancer cells.^[37,38]^ In this study, the TISIDB database was used to analyze the correlation between the mRNA expression level of SQLE and the expression of immunostimulators in sarcoma. The results showed that the mRNA expression level of SQLE was negatively correlated with the expression of CD160 and CD244. CD160 is a glycoprotein expressed on the surface of various immune cells, including cytotoxic T cells, natural killer (NK) cells, and subsets of Tregs.^[39]^ CD160 has been shown to regulate the activation, proliferation, and cytotoxicity of immune cells through its interaction with classical and nonclassical major histocompatibility complex molecules^[40]^ In the context of cancer, CD160 can promote antitumor immunity by enhancing the cytotoxic activity of T cells and NK cells. However, it can also contribute to immune evasion by promoting the suppressive function of Tregs.^[41]^ CD244, also known as 2B4, is a transmembrane protein mainly expressed in NK cells and some subsets of T cells. It functions as both an activating and inhibitory receptor, depending on the signaling adaptors associated with its cytoplasmic tail.^[42]^ In cancer, CD244 can either promote or suppress antitumor immunity, depending on the balance of activating and inhibitory signals. An elevated expression of CD244 has been reported in various cancers, including sarcoma, and is often associated with a poor prognosis.^[43]^ The observed negative correlation between SQLE and both CD160 and CD244 suggests that SQLE overexpression in sarcoma may lead to a reduced expression of these immune checkpoint molecules, potentially impairing antitumor immune responses mediated by T cells and NK cells.^[44]^ Alternatively, SQLE overexpression might enhance the suppressive function of Tregs, further contributing to immune evasion.^[16]^ Further experimental studies are warranted to elucidate the precise molecular mechanisms underlying the interplay between SQLE, CD160, and CD244 in sarcoma.

Immunoinhibitors, on the other hand, are substances that suppress the immune system’s response. These substances can include checkpoint inhibitors, which are antibodies that block proteins that normally inhibit the immune system’s response to cancer cells.^[41]^ The results showed that the mRNA expression level of SQLE was negatively correlated with the expression of C10orf54 and TNFRSF14. C10orf54, also known as V-domain Ig suppressor of T cell activation (VISTA), is an immune checkpoint molecule expressed on the surface of various immune cells, including T cells, macrophages, and myeloid-derived suppressor cells (MDSCs). VISTA has been reported to inhibit T cell activation, proliferation, and cytokine production by interacting with its ligands on antigen-presenting cells or T cells.^[45]^ In cancer, VISTA has been shown to promote immune evasion by suppressing antitumor T cell responses, making it an attractive therapeutic target for immune checkpoint blockade.^[46]^ TNFRSF14, also known as herpesvirus entry mediator (HVEM), is a member of the tumor necrosis factor receptor superfamily and is expressed on the surface of various immune cells, including T cells, B cells, and dendritic cells. HVEM interacts with multiple ligands, including LIGHT and BTLA, to regulate immune cell activation, survival, and differentiation.^[47,48]^ In the context of cancer, HVEM has been reported to play a dual role in immune regulation, either promoting antitumor immunity by enhancing T cell activation and function or contributing to immune evasion by promoting the suppressive activity of regulatory T cells (Tregs).^[49]^ The observed negative correlation between SQLE and both C10orf54 and TNFRSF14 suggests that SQLE overexpression in sarcoma may lead to a decreased expression of these immune checkpoint molecules, potentially affecting immune regulation within the TME. This could result in the impairment of antitumor immune responses mediated by T cells and other immune cells, or the enhancement of immune suppressive mechanisms mediated by Tregs, MDSCs, or other immune cell subsets. Further studies are needed to elucidate the precise molecular mechanisms underlying the interaction between SQLE, C10orf54, and TNFRSF14 in sarcoma. Understanding these relationships may inform the development of novel therapeutic approaches that target not only SQLE-mediated cholesterol biosynthesis but also immune checkpoint modulation, potentially improving the clinical outcomes of sarcoma patients.

Chemokines and their receptors play an important role in the directed migration of immune cells.^[50]^ In this study, the TISIDB database was used to analyze the correlation between the mRNA expression level of SQLE and the expression of immune cell chemokines and chemokine receptors in sarcoma. The results showed that the mRNA expression level of SQLE was negatively correlated with the expression of CCL19, CX3CL1, CCR2, and CCR7, suggesting that the mRNA expression of SQLE may inhibit the migration of immune cells to the TME. CCL19, a chemokine ligand, is involved in the recruitment and activation of various immune cells, including DCs, T cells, and B cells. It primarily interacts with its receptor CCR7 to regulate immune cell homing to lymph nodes and secondary lymphoid organs, as well as the formation of immunological synapses. In the context of cancer, CCL19 has been reported to promote antitumor immunity by facilitating immune cell infiltration into the TME and enhancing T cell activation.^[51–53]^ CX3CL1, also known as fractalkine, is a unique chemokine that exists both as a soluble molecule and as a membrane-bound protein. It interacts with its receptor, CX3CR1, to regulate the migration, adhesion, and activation of various immune cells, including monocytes, T cells, and NK cells. In cancer, CX3CL1 has been shown to modulate antitumor immunity by promoting the recruitment and cytotoxic activity of NK cells and CD8^+^ T cells.^[54,55]^ CCR2 is a chemokine receptor primarily expressed on monocytes, macrophages, and subsets of T cells. It binds to its ligands, CCL2 and CCL7, to mediate immune cell migration and infiltration into tissues. In cancer, CCR2 has been implicated in the recruitment of tumor-associated macrophages and MDSCs, which can promote tumor growth and immune evasion.^[56,57]^ CCR7, as mentioned earlier, is the primary receptor for CCL19 and is expressed in various immune cells, including T cells, B cells, and DCs. CCR7 is involved in immune cell trafficking to lymph nodes and secondary lymphoid organs and plays a crucial role in the initiation of adaptive immune responses. In the context of cancer, CCR7 has been reported to play a dual role, either promoting antitumor immunity by enhancing T cell activation or facilitating immune evasion by promoting lymphatic metastasis.^[58]^ The observed negative correlation between SQLE and CCL19, CX3CL1, CCR2, and CCR7 suggests that SQLE overexpression in sarcoma may lead to a reduced expression of these chemokines and chemokine receptors, potentially affecting immune cell recruitment and infiltration in the TME. This could result in an impaired antitumor immune response or the enhancement of immune suppressive mechanisms mediated by tumor-associated macrophages, MDSCs, or other immune cell subsets. Further studies are needed to elucidate the precise molecular mechanisms underlying the interaction between SQLE and these chemokines and chemokine receptors in sarcoma. Understanding these relationships may inform the development of novel therapeutic approaches that target not only SQLE-mediated cholesterol biosynthesis but also chemokine-mediated immune cell trafficking and infiltration, potentially improving the clinical outcomes of sarcoma patients.

In conclusion, our study found that the expression of SQLE was significantly upregulated and closely related to the poor prognosis of sarcoma patients. SQLE has a certain reference value for the diagnosis and prognosis of sarcoma. SQLE may affect sarcoma progression through immune infiltration. It could serve as a novel pre-marker for sarcoma patients.

However, even though we systematically analyzed SQLE and performed cross-validation using different databases, this study has its limitations. First, the role of the SQLE in sarcoma may vary in the reproducibility of microarray data generated by different laboratories. Second, we need in vivo/in vitro experiments to demonstrate the effect of SQLE on sarcoma to increase the reliability of our results. Third, our results show that the AUC area of SQLE is 0.903, indicating that SQLE has a good diagnostic effect on esophageal cancer. At present, the relevant experiments of detecting SQLE in peripheral blood and liver tissues are in progress, and the relevant research results will be published in the follow-up research reports. Finally, although we concluded that SQLE is closely associated with immune infiltration and prognosis in sarcoma, we lack direct evidence that SQLE affects prognosis through its involvement in immune infiltration. We will further explore these questions in future experiments.

## Author contributions

Data curation: Mengwei Shao, Mingbo Wang, Xiliang Wang.

**Formal analysis:** Mengwei Shao, Mingbo Wang.

**Writing – original draft:** Mengwei Shao, Mingbo Wang, Xiliang Wang, Xiaodong Feng.

**Validation:** Xiliang Wang, Xiaodong Feng.

**Software:** Xiaodong Feng.

**Supervision:** Lifeng Zhang, Huicheng Lv.

**Writing – review & editing:** Huicheng Lv.
